# 

**Published:** 1867-01

**Authors:** 


### CONTENTS FOR JANUARY.

ORIGINAL CONTRIBUTIONS.

Fifteen Cases of "Cerebro-Spinal Meningitis," and their Post Mortem Appearances. Presented by Chas. M. Clark, M.D., late Surgeon 39th Ill. Vol. Infantry, and Chief Operating Surgeon 24th Army Corps, ... 1

Introduction to the Course of Lectures on Diseases of the Eye, in Rush Medical College, Session of 1866--7. By E. L. Holmes, M.D., Surgeon to the Chicago Charitable Eye and Ear Infirmary, etc., ... 14

PROCEEDINGS OF MEDICAL SOCIETIES.

Chicago Medical Society, ... 19

Pathological Specimens of the Heart—Sulphuric Acid in Variola and Erysipelas—Use of a Concave Reflector in Examination of the Ear—Case of Cerebro-Spinal Meningitis—Elgin Milk Condensing Company.

Military Tract Association, ... 26

Report of Cases, and Presentation of Morbid Specimens.

Proceedings of the Esculapian Society of the Wabash Valley, ... 29

Morgan County Medical Society, ... 31

SELECTED ARTICLES, ... 34

Hysterical Mania, caused by Irritation of Spicula of Bone—Treatment of Chronic Metritis—Ethereal Pyrotechnics—Hypodermic Treatment of Diseases.

EDITORIAL, ... 41

*Book Notices.*—The Principles and Practice of Medicine—Notes on Epidemics: For the Use of the People—A Manual of Auscultation and Percussion.

BOOKS AND PAMPHLETS RECEIVED, ... 42

Opinion Relative to the Testamentary Capacity of the late James C. Johnston—Transactions of the Medical Society of the State of Pennsylvania—Report on the Sanitary Relations of the State of Kansas—Report on the Climatology of Kansas—Infantile Paralysis and its Attendant Deformities—Messages and Documents, for 1855-6—Report of the Surgeon-General—Atlantic Monthly—The Riverside Magazine for Young People.

City Mortality, ... 47

Mortality of the Year, ... 48

Errata, ... 48

